# Burkitt Lymphoma: An Atypical Presentation and Dismal Outcome

**DOI:** 10.7759/cureus.90084

**Published:** 2025-08-14

**Authors:** Inês M Araújo, Marta Viana-Pereira, Inês Silveira, Soraia G Araújo, Ana Oliveira

**Affiliations:** 1 Internal Medicine, Unidade Local de Saúde de Braga, Braga, PRT; 2 Medical Oncology, Unidade Local de Saúde de Braga, Braga, PRT; 3 Intensive Care Unit, Unidade Local de Saúde de Braga, Braga, PRT

**Keywords:** burkitt lymphoma, diagnostic challenge, ebv infection, leptomeningeal invasion, non-hodgkin lymphoma

## Abstract

Burkitt lymphoma (BL) is a highly aggressive B-cell non-Hodgkin lymphoma that manifests more frequently in children than adults. Disease presentation in adults includes abdominal masses, B symptoms, tumor lysis, and central nervous system involvement, mainly in the leptomeninges. The central nervous system involvement is a common complication of this type of lymphoma, but it is unusual at presentation. The authors present the diagnostic challenges and therapeutic approach of a complex case of a 55-year-old man presenting with symptoms of spinal cord compression at diagnosis, an atypical presentation of Burkitt lymphoma.

## Introduction

Burkitt lymphoma (BL) is a highly aggressive B-cell non-Hodgkin lymphoma (NHL) whose hallmark is the translocation and dysregulation of the MYC gene [1]. It is common in pediatric age and accounts for only 1-2% of NHL cases in adults [1]. There are three subtypes of BL recognized by the World Health Organization (WHO): endemic, sporadic, and immunodeficiency-associated disease [2]. In the adult population, the involvement of the central nervous system (CNS) and bone marrow at diagnosis is reported to be around 13-19% and 30-38%, respectively [2,3]. However, the involvement of the CNS as a complication of BL reaches 40% in some studies [4]. It may involve the meninges, spinal cord, cranial nerves, or even the brain parenchyma, leading to focal neurological deficits or convulsions. The authors present the diagnostic challenges and therapeutic approach of a complex case of BL with spinal involvement as the first manifestation of the disease.

## Case presentation

A 55-year-old male patient, without known prior medical conditions, was evaluated in the emergency department for intense low back pain lasting one month. Furthermore, he reported paresthesias and progressive loss of strength in the lower limbs and right hand, over the last two weeks, which progressed to the point of being unable to walk independently. Other symptoms included constipation and urinary retention. When questioned, he also reported intermittent fever over the last two weeks, night sweats, and loss of 4 kg over one month.

**Figure 1:**
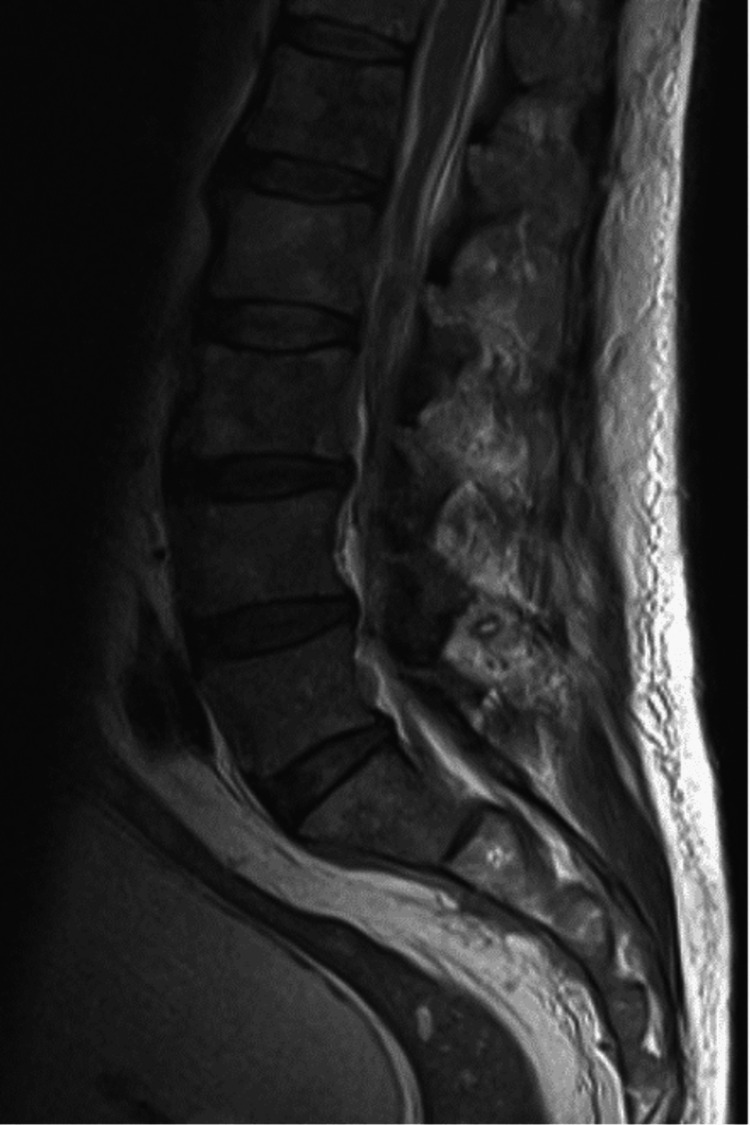
Magnetic resonance imaging performed in the emergency department suggesting bone marrow hyperplasia or tumor infiltration.

The neurologic assessment showed flaccid paraparesis in the lower limbs (grade 2 according to the Medical Research Council strength scale) and decreased reflexes, without proprioception deficits. There was a distal motor deficit in the right arm (grade 3 in the Medical Research Council strength scale). The laboratory workup revealed anemia, the presence of atypical lymphocytes without lymphocytosis, and increased C-reactive protein levels (Table 1). Cranioencephalic computed tomography (CT) excluded signs of acute hemorrhagic/ischemic injuries, abscess, or expansive brain lesions, and spine CT revealed only degenerative disease, which did not correspond to the patient's symptoms. Magnetic resonance imaging (MRI) was performed for better characterization, showing a hypointense signal on T1 and a mild hyperintense signal on STIR, which could translate into bone marrow hyperplasia or tumor infiltration (Figure 1). The patient was admitted for investigation and treatment. 

**Table 1:** Initial laboratory workup MCV: mean corpuscular volume; MCHC: mean corpuscular hemoglobin concentration; ESR: erythrocyte sedimentation rate; AST: aspartate aminotransferase; ALT: alanine aminotransferase; ALP: alkaline phosphatase; LDH: lactate dehydrogenase; TSH: thyroid-stimulating hormone

Laboratory workup parameters	Values	Reference values
Hemoglobin	9.50 g/dL	13.5-17 g/dL
MCV	86.60 fL	81.8-95.5 fL
MCHC	32.80 g/dL	32.4-35 g/dL
Leukocytes	9700 /uL	4.0-11.0 /µL
Neutrophils	4000 /uL	1.8-7.1 /µL
Lymphocytes	3200 /uL	1.2-3.4 /µL
Monocytes	800 /uL	0.2-0.9 /µL
Metamyelocytes	500 /uL	
Myelocytes	800 /uL	
Atypical lymphocytes	400 /uL	
Platelets	240000 /uL	150-400 /µL
Folic acid	3.20 ng/mL	>5.38 ng/mL
Vitamin B12	334 pg/mL	211-911 pg/mL
ESR	96 mm/h	1-15 mm/h
Urea	45 mg/dL	10-50 mg/dL
Creatinine	0.60 mg/dL	0.6-1.1 mg/dL
Potassium	4.10 mmol/L	3.5-5.5 mmol/L
Sodium	135 mmol/L	135-145 mmol/L
Total bilirubin	0.32 mg/dL	0.3-1.3 mg/dL
AST	108 U/L	12-40 U/L
ALT	64 U/L	7-40 U/L
ALP	232 U/L	46-116 U/L
LDH	1187 U/L	120-246 U/L
Haptoglobin	280 mg/dL	40-280 mg/dL
C-reactive protein	183.10 mg/L	<5 mg/L
Procalcitonin	0.03 ng/mL	<0.05 ng/mL
TSH	1.01 uUI/mL	0.55-4.78 µIU/mL

During hospitalization, fever and neurologic symptoms were persistent. The result of the serology for human immunodeficiency virus was negative. The was previous contact with hepatitis B virus and cytomegalovirus, and positive serology for Epstein-Barr virus (EBV) with positive viral load (Table 2).

**Table 2:** Infectious evaluation HIV: human immunodeficiency cirus; HBsAg: Hepatitis B surface antigen; Anti-HBs: hepatitis B surface antibody; HBeAg: hepatitis B e antigen; Anti-HBe: hepatitis B e antibody; HBcAb: hepatitis B core antibody; HBV DNA: hepatitis B Virus DNA; CMV: cytomegalovirus; EBV: Epstein-Barr virus; Anti-EBV VCA: Anti-EBV viral capsid antigen; VDRL: venereal disease research laboratory; THPA: treponema pallidum hemagglutination assay

Infectious disease parameters	Results
HIV I/II	Negative
Hepatitis B	
HBsAg	Negative
Anti-HBs	Positive
HBeAg	Negative
Anti-HBe	Positive
HBcAb	Positive
HBV DNA	Negative
Hepatitis C (anti-HCV)	Negative
CMV	
Anti-CMV IgM	Negative
Anti-CMV IgG	Positive
EBV	
EBV early antigen	Positive (>150 U/mL)
Anti-EBV VCA IgG	Positive (>750 U/mL)
Anti-EBV VCA IgM	Positive (>160 U/mL)
EBV nuclear antigen	Positive (>600 U/mL)
EBV DNA	Positive
Syphilis	
VDRL	Negative
THPA	Negative

Lumbar puncture (LP) was performed, revealing increased cellularity with predominance of lymphocytes/monocytes and marked proteinorrhachia. During the myelogram and bone marrow biopsy, the appearance of purulent content raised the hypothesis of miliary tuberculosis, as there was an epidemiological context. Empiric treatment of tuberculosis was then initiated simultaneously with prednisolone 60 mg, given the hypothesis of lymphoproliferative disorder. Nevertheless, the search for bacteria, fungi, or mycobacteria was negative both in the biopsies and blood (by cultural method and polymerase chain reaction). The LP cytology was suggestive of a lymphoproliferative process. Upon having these results, antibacillary therapy was suspended. 

**Figure 2:**
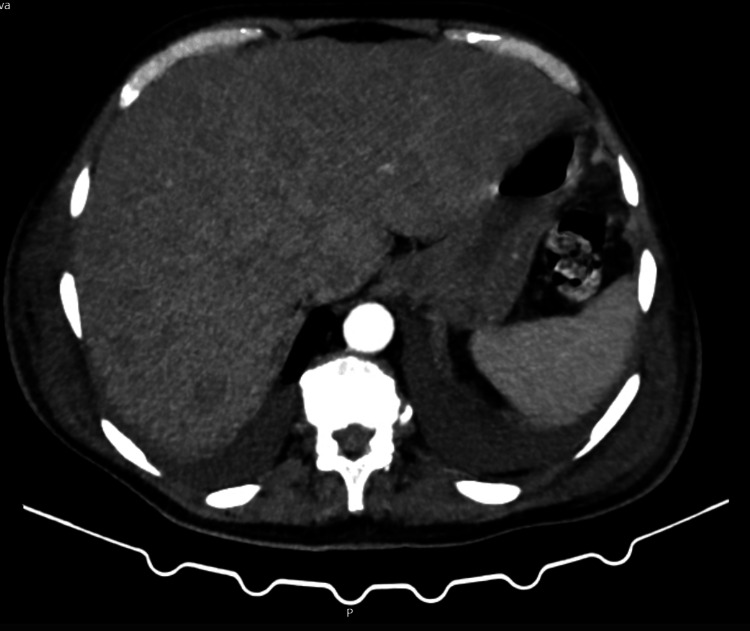
Thoraco-abdomino-pelvic CT revealed an enlarged liver with multiple nodular formations.

**Figure 3:**
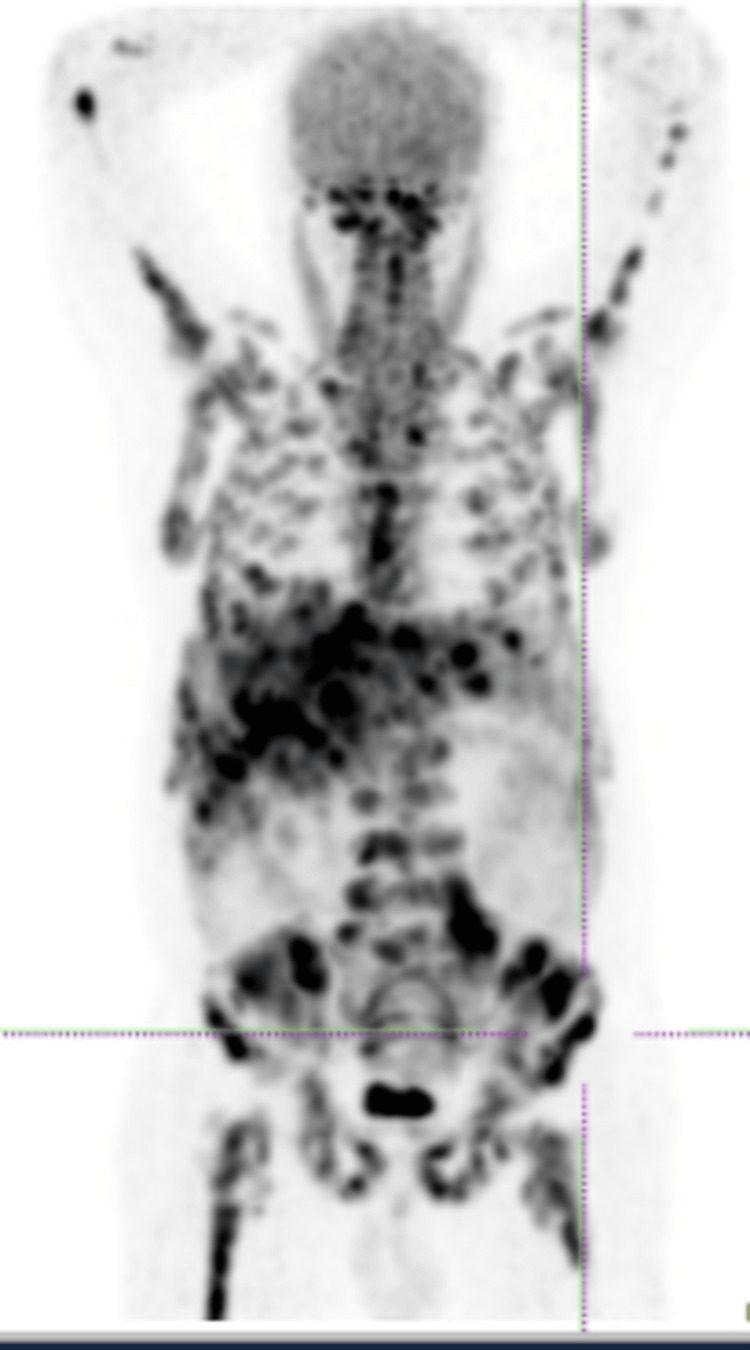
Positron-emission tomography showed diffuse bone/medullary and hepatic infiltration due to high-grade metabolic pathology

Thoraco-abdomino-pelvic CT revealed an enlarged liver with multiple nodular formations that were biopsied afterwards (Figure 2). Positron-emission tomography showed diffuse bone/medullary and hepatic infiltration due to high-grade metabolic pathology (Figure 3). 

**Figure 4:**
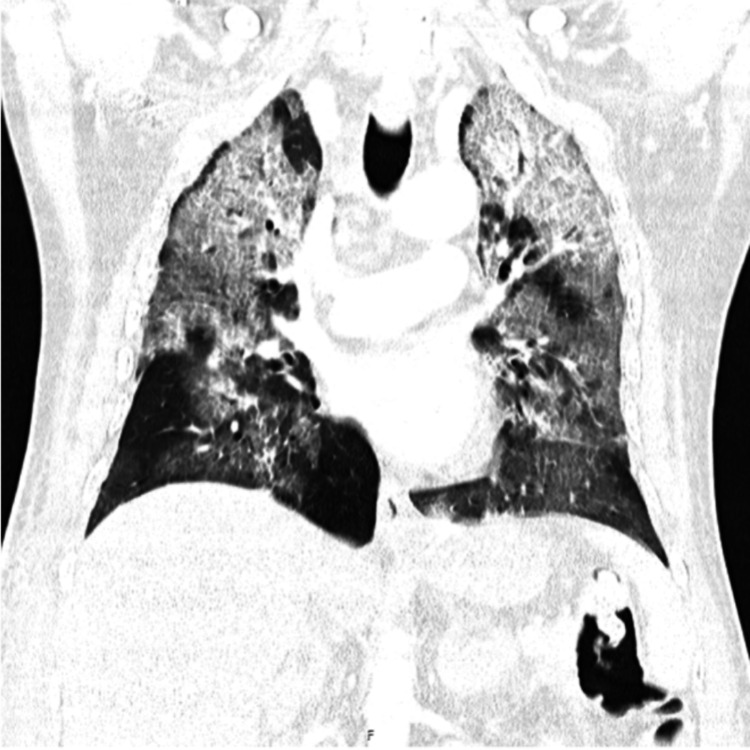
Thoracic tomography reveals extensive areas of densification of the lung parenchyma bilaterally, sparing the subpleural planes, showing a crazy-paving pattern in the upper lobes, suggestive of acute respiratory distress syndrome.

On Day 16 of hospitalization, the patient developed a severe respiratory failure, leading to an acute respiratory distress syndrome (ARDS) with multiorgan failure, requiring admission to an intensive care unit (Figure 4). A medullary and liver biopsy carried out on the same day yielded the diagnosis of large cell B lymphoma, with an immunohistochemical profile compatible with BL. Broad-spectrum antibiotic therapy was initiated, as well as life-saving chemotherapy with a CHOP regimen as recommended by the Hematology. Despite the established therapy, the patient died the following day.

## Discussion

Three distinct forms of BL are recognized with similar aggressive behavior: endemic, sporadic, and immunodeficiency-associated. The endemic form is found in Africa and New Guinea and is more prevalent in children [5]. Sporadic form is more common among Caucasian patients with a median age at diagnosis of 30 years [6]. Both clinical forms are more prevalent in male patients [7]. The immunodeficiency-associated variant is almost always found in patients with HIV infection, typically around 40 years of age, and less commonly in patients with other causes of immunodeficiency. Its distribution is comparable between both genders [1].

BL rarely affects the CNS as a primary disease, with only a few cases of primary CNS BL reported in recent years [8]. Conversely, secondary CNS involvement by BL can be as high as 50%; hence, these populations benefit from CNS prophylaxis [9]. Nevertheless, spinal and leptomeningeal disease involvement in the absence of parenchymal brain disease is rare. This case is particularly relevant due to its atypical presentation. The CNS involvement was the first involvement to be noted, even before the appearance of B symptoms, confirming the diagnostic challenge of this case and providing an example of an approach to this type of unusual presentation. 

Also, CNS involvement by lymphoma is particularly common among immunocompromised patients, although the incidence in the immunocompetent elderly population is slowly increasing [8]. Our case is again particularly unusual as our patient did not fall into any of these risk groups.

BL is known to be associated with EBV, and, although the exact mechanism underlying this connection remains unclear, studies suggest that EBV infection might confer a survival advantage for the neoplastic cells [5]. Chronic EBV infection appears to play a role in virtually all cases of endemic BL, but it can also be present in the immunodeficiency-associated and sporadic forms, as seen in this case. 

A high lactate dehydrogenase (LDH) value is associated with more aggressive disease and a worse prognosis. Treatment for this type of lymphoma is based on intensive chemotherapy regimens and includes three stages: induction, consolidation, and maintenance [2,10]. The choice of starting chemotherapy with the CHOP regimen was due to concerns for excessive toxicity with a more dose-intensive regimen in this particular situation, due to the extent of the disease and the development of acute kidney injury.

## Conclusions

This case illustrates a rare and atypical presentation of BL with central nervous system involvement in an immunocompetent adult. The absence of BL symptoms at the beginning and common risk factors (such as immunodeficiency) made diagnosis even more challenging, emphasizing the need for clinical awareness in similar scenarios.

When suspected, BL diagnosis should be accelerated in order to allow a prompt treatment due to the nature of the disease, as highlighted by the rapid and adverse evolution of the presented case.
